# Using Risk Communication Strategies for Zika Virus Prevention and Control Driven by Community-Based Participatory Research

**DOI:** 10.3390/ijerph15112505

**Published:** 2018-11-09

**Authors:** Deborah Juarbe-Rey, Adriana Obén Pérez, Roberto Papo Christian P. Santoni, Melissa Ramírez Ramírez, Mildred Vera

**Affiliations:** 1 Center for Evaluation and Sociomedical Research, Graduate School of Public Health, University of Puerto Rico, Medical Sciences Campus, P.O. Box 365067, San Juan, PR 00936-5067, USA; deborah.juarbe1@upr.edu (D.J.-R.); adriana.oben@upr.edu (A.O.P.); melissa.ramirez2@upr.edu (M.R.R.); 2 Community Leader, Manuel A. Pérez Public Housing, Puerto Rico, San Juan, PR 00936-5067, USA; santoni.rp@gmail.com; 3 Department of Health Services Administration, Graduate School of Public Health, University of Puerto Rico, Medical Sciences Campus, P.O. Box 365067, San Juan, PR 00936-5067, USA

**Keywords:** Zika, community-based participatory research, risk communication

## Abstract

*Background:* In this study, we use community-based participatory processes to engage community and academic partners in a meaningful exchange to identify and pilot test risk communication strategies for Zika virus prevention and control. *Methods:* Community members were actively involved in planning, developing, and implementing a risk communication initiative. Qualitative and quantitative data gathered through individual interviews, focus groups, and community advisory board input provided information for the identification of relevant risk communication strategies to increase the understanding about Zika virus and to promote behavior change. To examine its impact we obtained baseline and follow-up data from a random sample of 75 community residents. A face-to-face interview was conducted to assess community members’ knowledge, attitudes, and behaviors regarding Zika virus infection. *Results:* Study activities focused on three risk communication strategies: Zika awareness health fair, health education through theater, and community forums and workshops. The risk communication initiative was implemented over a two-month period. Findings from baseline and follow-up data demonstrated significant positive changes in respondents’ recognition of personal and community responsibility for the prevention of Zika infection, increased knowledge of prevention strategies, and enhanced engagement in preventive behaviors for mosquito control. *Conclusion:* Our findings sustain the benefits of community based participatory research for the design and implementation of risk communication strategies that are relevant to enable residents in low-income communities to take informed decisions for the protection against Zika virus and other mosquito-borne diseases.

## 1. Introduction

Mosquito-borne diseases are common in Puerto Rico and represent a significant public health challenge. The Zika virus is the most recent mosquito-borne disease outbreak to impact the Island. Its main transmission vector is the Aedes aegypti mosquito, which also spreads dengue and chikungunya viruses. Mosquito-borne diseases are expected to become a bigger problem in a warming world since weather conditions affect their emergence [1,2,3,4,5]. Zika infection is usually a mild illness and most people do not report symptoms. However, an increased risk of neurologic complications, like Guillain-Barré syndrome, and microcephaly are associated with Zika virus infection [6,7,8]. Children born with microcephaly are likely to experience developmental, intellectual, physical, and sensory impairments throughout their lifetime [9,10,11]. Since there is no treatment or vaccine available, WHO recommendations focus attention on the Aedes mosquito as the main vector for Zika infection [12]. They highlight that the most important protective measures are the control of mosquito populations and the prevention of mosquito bites.

Effective risk communication and community engagement strategies are essential for enabling people to make informed decisions for protection against mosquito-borne diseases, such as the Zika virus. Risk communication activities consisting mainly of the dissemination of information about health risks and behavior recommendations to reduce those risks have frequently been unsuccessful [13]. Significant changes have taken place among community residents over the past years. Foremost among these is that experts and officials are less trusted. WHO highlights that risk communication needs to be approached from the perspective of shared, multi-directional communications, and engagement with affected populations [13,14].

Lower-income residents are at increased exposure to mosquito bites and pregnant women bear a greater risk of complications from Zika virus infection. However, it has been found that public health promotion activities have not been successful in increasing awareness about the Zika virus infection and engaging them to adopt behaviors geared at the mitigation of risks [15,16,17]. The American Public Health Association urges that more comprehensive and participatory approaches to public health research and practice are essential [18]. Furthermore, they highlight the benefits of community based participatory research as a promising approach for building connections between academics and communities.

Community based participatory research (CBPR) is defined as “a collaborative approach to research that equitably involves all partners in the research process and recognizes the unique strengths that each brings. CBPR begins with a research topic of importance to the community and has the aim of combining knowledge with action and achieving social change to improve health outcomes and eliminate health disparities” [19]. Emphasis is placed on the relevance of partnerships between researchers and community members in the development, implementation, and evaluation of the research [20,21]. Over the past years, the use of community-based participatory research approaches has increased, contributing to address health disparities by adapting interventions within local contexts, reinforcing local cultural knowledge, easing mistrust, and training investigators from communities that experience the health disparities [22,23,24,25,26]. In the study described here, we used community-based participatory processes to develop strategic partnerships among community and academic groups for planning and pilot testing risk communication and community engagement strategies for Zika virus prevention. Specifically, we examine the process and outcomes of a risk communication initiative to increase Zika virus awareness and health enhancing behaviors among residents of a low-income housing project. Study findings could establish an evidence base for expanded research that has the potential to shift current risk communication practices.

## 2. Materials and Methods

In this section, we describe two phases of our CBPR study, the action steps that took place during each study phase, as well as the data collection and analysis strategies used. Although the steps are discussed sequentially, they are more circular than linear. The design and methods of this project were developed within the partnership, informed by the CBPR approach [27]. All research related to this study was approved by the institutional review board at the Medical Sciences Campus of the University of Puerto Rico (NO. B1060116).

### 2.1. Phase 1 Formative

#### 2.1.1. Establishing the Community-Academic Partnership

The process of partnership formation for this CBPR study evolved from conversations between a key community leader of the Manuel A. Perez (MAP) housing project and investigators from the Graduate School of Public Health (GSPH), Medical Sciences Campus, University of Puerto Rico. Within CBPR, successful community-academic partnerships are fostered through attention to networking, cooperation, collaboration, and shared decision-making [28,29]. During the early stages of forming this partnership, our meetings centered on exploring mutual research interests about Zika virus prevention and building relationships, allowing partners to develop a sense of engagement and ownership. After holding meetings for several months, a CBPR project focused on increasing Zika virus awareness, engagement, and mobilization was conceptualized and eventually funded for a 12-month period.

Two levels of project organization facilitated the involvement of all partners in the research process, the study team, and the community advisory board. The study team attended the needs of the project and provided support to the community advisory board. Its members included the community partner, academic investigators, and study staff. The study team held weekly meetings to coordinate initiatives, plan future activities, and evaluate study development. The community advisory board included five representatives from local community sectors, including women in reproductive age, mothers, sport leaders, students, and community leaders. They were instrumental in: (1) providing guidance on the most effective ways to conduct research in the community, (2) making sure the project addressed community needs, and (3) helping interpret findings. Board members met once or twice a month and received a small stipend for their participation and work.

#### 2.1.2. Assessing Community Strengths and Dynamics

The CBPR approach sustains that problems that impact a community are best understood by its members and that communities should be directly involved in research that address these [30]. To assess community strengths and dynamics that are related to Zika virus infection, four focus groups were conducted with diverse community groups: adult females in reproductive age, pregnant females, adolescent females, and adolescent males. Community partners were responsible to reach out and recruit community members. A total of 25 MAP females (*n* = 19) and males (*n* = 6) were recruited. Focus groups were conducted in the MAP nursing center, a venue considered to be convenient and widely accepted by the community. A health professional with qualitative expertise moderated discussions. Members of the academic-community partnership developed a facilitator guide that included the areas to be discussed by the groups and probes that could contribute to direct the discussion and generate the information needed to address the study focus and aims. Thematic framework analysis was used to extract and classify important themes from focus groups and key informant interviews [31,32]. This process consisted of several steps: transcribing the audiotaped interviews, reading the transcription to become familiar with the data; applying codes to relevant passages; and, meeting between reviewers to group similar codes into categories, applying the categories to subsequent transcripts, summarizing the data by category, and interpreting the data. Findings provided us with a richer and deeper understanding of community assets and Zika prevention and control practices identified as acceptable to MAP residents. Relevant findings included: (1) strong commitment among community residents towards adoption of Zika prevention and control practices; (2) limitations associated with prevention practices (households have no screens, warm weather and lack of air conditioning limit disposition to use mosquito nets and covering clothing, economic barrier to household members’ daily use of mosquito repellent); and, (3) practices with high receptivity among community members (reduction of sites where mosquitos can breed by emptying, cleaning, covering, or discarding containers). Results were particularly effective in supplying information about community members understanding and perceptions regarding Zika virus infection and communication strategies identified as relevant and acceptable to MAP residents.

#### 2.1.3. Description of Communication Strategies

The next step of the formative phase involved identifying appropriate approaches to provide educational information and supportive resources to engage MAP community members in the prevention of Zika virus infection. Qualitative and quantitative data obtained from community members and the community advisory board input guided the selection of three risk communication strategies: Zika awareness health fair, health education through theater, and community forums. Relevant criteria for the selection of these strategies was that they could attract large numbers of community members who could benefit from information about Zika infection, generate a sense of partnership within the community, and enhance communication among community members and health promoters. All members of the community were invited to participate in the health fair, theater production, and community forums through the distribution of flyers, home visits, and the promotion of the events while using loudspeakers.

The first strategy, Zika awareness health fair, provided education about Zika infection and prevention practices. The planning committee included community and academic researchers, community members, and representatives of the MAP management “MAS Corporation”. Periodic meetings were held to update partners, coordinate efforts, examine publicity plans, distribute responsibilities, and identify needs for the day of the event. Contributions from focus groups and community advisory board members were taken into consideration in planning and implementing the Zika awareness health fair, these included: (1) identifying a highly visible location, (2) setting a festive mood with balloons and music, (3) providing popular food and beverages, (4) selecting a well-recognized TV personality as master of ceremony, and (5) identifying event hosts with our project name “Unidos Contra el Zika”. The aim was to provide entertainment in a pleasant atmosphere while developing an interest in learning about Zika virus and strategies for vector control.

At the Zika awareness health fair, community members were encouraged to explore at their own pace health exhibits. Educational materials were distributed. Participants were able to ask health-related questions and receive further information. Also, musical sessions were combined with educational capsules at which the master of ceremony interviewed health professionals and community members who talked about strategies for vector control and experiences that are related to Zika virus. Giveaways were distributed. In addition, for each exhibit or information table, a gift card was raffled among community members that attended.

The second strategy involved health education through theater. The assessment of community assets in focus group discussions revealed that performances of the MAP community youth drama club were very well received by the community. The drama club is an after school program that presents performances to the community on a regular basis. After several meetings between the club director and community advisory board members, agreement was reached on the usefulness of a theatrical performance to convey information about Zika infection risks and preventive behaviors for mosquito control. The club director wrote a play script addressing this topic. To increase the relevance for the MAP community, the script was inspired by a community member’s Zika related experience with Guillain-Barré syndrome. After the performance the community member who experienced the syndrome and a physician held a question and answer session with the audience.

The third strategy, community forums focused on generating a sense of partnership within the community to adopt behaviors that reduce Zika health risks. WHO recommendations for Zika virus infection indicate the need for the reduction of sites where mosquitos can breed [33]. Specifically, they highlight that “families and the general community … be actively involved in eliminating mosquito breeding sites from their homes” [34]. After initial activities, community forums were held to facilitate the exchange of views about Zika virus infection and mosquito control activities. Discussions were led by facilitators with experience in CBPR community mobilization principles and Zika infection. Two forums were organized, one with adult and adolescent MAP residents and another with members of the basketball community team.

### 2.2. Phase 2 Conducting the Research

#### 2.2.1. Setting and Area Served

This pilot study was conducted in the Manuel A. Perez public housing project. MAP is located in Rio Piedras, Puerto Rico and is one of the largest public housing projects in the Island. It is divided in three sectors and has approximately 2300 adult residents that are distributed in 1962 housing units. One MAP sector, which includes approximately 1100 adults distributed in 850 housing units, was included in this study.

#### 2.2.2. Study Activities

Study activities focused on increasing Zika virus awareness, engagement, and mobilization using the risk communication strategies previously discussed. The activities were implemented over a two-month period. The Zika awareness health fair was convened a Sunday afternoon at a central location in MAP. It was attended by 140 community members. MAP residents in the study sector were invited to a theatrical performance that was held the following week. This activity was attended by 130 community residents. The community forums were held over the following weeks with the participation of 65 MAP residents.

#### 2.2.3. Data Collection

To examine the impact of the communication strategies pilot tested, we obtained baseline and follow-up data from a random sample of community residents. Previous to the implementation of study activities, a face-to-face interview was conducted to assess community members’ knowledge, attitudes, and behaviors regarding Zika virus. After study activities were conducted, the same residents were contacted for a follow-up interview to assess the same variables as the initial assessment. During the recruitment of the community sample, community partners visited MAP neighborhoods to provide information about the “Unidos Contra el Zika” project. Trained interviewers were responsible for the administration of face-to-face interviews that lasted about 30 min.

#### 2.2.4. Sample

The systematic random sampling technique was used to select the community sample for the baseline interview. This procedure involved recording all housing units in the study sector using standard listing techniques. A total of 121 units were selected. The first unit was selected randomly and thereafter every seventh unit was selected. Among the units included in the sample, 10 were empty and 19 did not have an eligible resident. During the baseline data collection, 77 of the units sampled yielded an interview. Only one participant refused to participate and 14 were not reached. The pre-assessment interview response rate was 84%. At each housing unit, an adult was contacted and explained the purpose of the project. The resident with the main responsibility for cleaning the household was identified as the potential respondent. If the potential respondent was not present, information about his/her schedule was obtained. Once contact was established with the potential respondent, the purpose of the project was explained. The interview was conducted if the potential respondent provided consent.

#### 2.2.5. Analysis

All of the variables were summarized with univariate analysis to confirm the accuracy and completeness of data. McNemar’s exact test for matched pairs was used to test changes in responses between the pre- and post-assessments. Statistical procedures were conducted using SPSS v24 (SPSS Inc., Chicago, IL, USA) at 0.05 *p*-value level of significance [35].

## 3. Results

In this section, we explore the potential outcomes of our CBPR pilot study. The impact of risk communication strategies was assessed using matched baseline and follow-up assessments. Out of 77 MAP residents who provided baseline data, 75 completed the follow-up interview, yielding a follow-up response rate of 97.4%. The mean age of those who completed both assessments was 43 years. Most (88%) were female, 22.7% had not completed high school, 54.2% were married, and 58.7% reported they were housewives.

The sources from which respondents indicated they received information about Zika is displayed in Table 1. Television programs was the most popular source of information in both assessments. While less than 3% of the respondents identified the community as a source of information about Zika in the pre-assessment, significant differences were observed at the post-assessment. Community activities (46.5%) and community handouts (29.6%) were identified by respondents as the second and fourth most common source of information about Zika virus infection. In the pre-assessment, family, friends, and neighbors were acknowledged as a source of information about Zika by approximately one-fifth of respondents. This proportion increased to almost one-third in the post-assessment, however the difference was not statistically significant. Findings also revealed a four-fold increase in the use of the internet as a source of information. In the pre-assessment, 4.2% reported using the internet, while the use of this source increased to 15.5% in the post-assessment. Few respondents acknowledged receiving information about the virus from a health provider, co-workers, or radio.

To assess changes in knowledge, respondents were asked to identify preventive behaviors. In the pre-assessment, from one-third to a half of the respondents reported mosquito control activities that included emptying and cleaning containers and the removal of standing water. In the post-assessment, from 60% to 70% of the respondents mentioned these activities. When asked to identify who is responsible for Zika prevention, in the pre-assessment 60% of the respondents identified that it was their responsibility. This proportion significantly increased to 85.3% in the post-assessment. Furthermore, the proportion of respondents that indicated community members were responsible for the prevention of Zika also increased significantly, from 14.7% in the pre-assessment to 38.7% in the post-assessment (Table 2).

In the post-assessment, 68% (*n* = 51) of the respondents indicated that they had taken preventive behaviors to avoid Zika virus infection. The practice of emptying and cleaning containers and removing standing water was reported by a significantly higher proportion of respondents at the post-assessment in comparison to the pre-assessment. While approximately one-third to a half of the respondents endorsed these practices in the initial assessment, almost three-fourths reported these practices at the post-assessment. Findings about the use of other protective behaviors revealed that two percent or less of the respondents acknowledged in the pre-assessment using: screens on windows and doors, mosquito net, condom, and covering clothing. No significant changes were observed in the follow-up data (Table 3).

## 4. Discussion

This pilot study provides valuable information about the process and outcomes of a risk communication initiative to increase Zika virus awareness and health enhancing behaviors among residents of the MAP housing project. Following CBPR principles, this study built upon having community and academic members collaborate as partners in the research process. While establishing the partnership, concerns were raised about previous experiences with academic researchers who collect data from the community for their own advancement with no benefits for community members. This situation, which has also been reported in other communities, is identified as “helicopter research”. This reference points at researchers who “fly” into the community, collect their data, and leave as soon as possible without further communication [28,36]. CBPR highlights as a guiding principle the need to “achieve a balance between knowledge generation and intervention for mutual benefit of all partners” [27]. Frequent meetings during this initial period allowed for addressing concerns and build trust among partners, work on a policy of open and honest communication, and demonstrate respectful collaboration in the research process.

Traditional health communication strategies rely on providing communities one-way messages, from experts to non-experts, with statistics or other scientific data and little consideration for beliefs, attitudes, or needs of the communities that they are addressing [37,38]. These practices have been found to have only weak or moderate impact [37]. Over the years, there has been an increasing recognition that risk communication involves a two-way exchange of information, in which engaging the community and addressing their particular concerns is as relevant as delivering key health messages [39,40]. The “Unidos contra el Zika” initiative is an example of a community engagement and risk communication project that applied an interactive process of information and opinion exchange to understand the concerns and meet the unique needs of the MAP community. Partnering with community members allowed for contextualizing risk communication strategies to convey health information in formats that were easily understood and well-received by community members. Furthermore, community members’ involvement in planning, developing, and implementing this risk communication initiative contributed to an increased sense of project ownership and empowerment as contributors in this research process. This pilot study also explored the outcomes of this initiative. Findings demonstrated significant positive changes in respondents’ recognition of personal and community responsibility for the prevention of Zika virus infection, increased knowledge of Zika virus prevention strategies, and enhanced engagement in preventive behaviors for mosquito control. Overall, these findings sustain the importance that health professionals take into consideration community-based participatory approaches for the design of risk communication and community engagement strategies that are essential to enable residents in low-income communities to make informed decisions for the protection against Zika virus and other mosquito-borne diseases.

There are several limitations to this study that should be taken into consideration in future research. First, establishing a community/academic partnership is a time-intensive undertaking that requires commitment at each phase from both community and academic members. Research efforts directed at fostering partnerships centered on community-based participatory processes need to take this into account when considering a timeframe for project development and implementation. Second, the current CBPR study was conducted in one low-income community. More research is necessary to assess the usefulness of this approach across different populations to identify risk communication and community engagement strategies that work best for each group, recognizing that these strategies may vary accordingly. Third, results pertaining to the impact of risk communication strategies on changes in knowledge and behaviors need to be interpreted with caution due to the absence of an appropriate control group. Finally, there are other risk communication and community engagement strategies for enabling people to make informed decisions for protection against mosquito-borne diseases that were not addressed in this study. Recent advancement in Internet-based technologies has resulted in the growth of infoveillance, which refers to the collation of health information from the internet for the purpose of surveillance and trending of emerging health crisis [41]. Previous studies demonstrated that the Internet can be used for data collection during an air-pollution crisis [42] and Facebook could be used in Zika outbreak communication [43]. Further research is warranted to evaluate the use of the Internet and social media for Zika virus prevention. Notwithstanding these limitations, the strengths of this study center in the use of CBPR processes for seeking guidance from the community to assess its unique needs for the design and implementation of risk communication strategies that are relevant and culturally appropriate.

## 5. Conclusions

Effective risk communication and community engagement strategies are essential for enabling people to make informed health decisions that will influence their overall well-being. Our findings sustain the benefits of community based participatory research for the design and implementation of risk communication strategies that are relevant to enable residents in low-income communities to take informed decisions for the protection against Zika virus and other mosquito-borne diseases.

## Figures and Tables

**Table 1:** Reported sources from which respondents received information about Zika at pre and post-assessments.

Sources of Information	Pre (*n* = 71)		Post (*n* = 71)		*p*-Value
	%	*n*	%	*n*	
**Where/From Whom Did You Received Zika Information?**					
Television	73.2	52	73.2	52	
Community activities	2.8	2	46.5	33	<0.001
Family, friends or neighbors	21.1	15	32.4	23	0.185
Community handouts	1.4	1	29.6	21	<0.001
Internet	4.2	3	15.5	11	0.057
Co-workers	5.6	4	4.2	3	1.00
Newspapers	8.5	6	18.3	13	0.092
Radio	8.5	6	1.4	1	0.125
Health center	12.7	9	4.2	3	0.109
Educational institution	7.0	5	8.5	6	0.756

**Table 2:** Respondent’s knowledge and attitudes regarding Zika prevention and vector control practices at pre and post-assessments.

Knowledge and Attitudes	Pre (*n* = 75)		Post (*n* = 75)		*p*-Value
	%	*n*	%	*n*	
Knowledge					
How can you prevent Zika in your house?					
Empty/clean water containers	49.3	37	70.7	53	0.009
Cover water storage containers	5.3	4	34.7	26	<0.001
Remove standing water	52.0	39	74.7	56	0.008
What can you do to prevent mosquito breeding sites?					
Empty/clean water containers	34.7	26	60.0	45	0.004
Remove standing water	49.3	37	73.3	55	0.008
Attitudes					
Who’s responsibility is it to prevent Zika?					
Personal responsibility	60.0	45	85.3	64	<0.001
Community members	14.7	11	38.7	29	<0.001

**Table 3:** Respondent’s preventive behaviors reported at pre and post-assessments.

Behaviors	Pre (*n* = 51)		Post (*n* = 51)		*p*-Value
	%	*n*	%	*n*	
What actions have you taken to prevent that you or relatives get Zika?					
Emptied/cleaned water containers	45.1	23	76.5	39	0.001
Removed standing water	37.3	19	72.5	37	0.001
Covered water containers	3.9	2	9.8	5	0.453
Used mosquito repellent	49.0	25	39.2	20	0.405
Sprayed or fumigated home	17.6	9	29.4	15	0.210
Used mosquito net	2.0	1		0	
Put screens on windows and doors		0	11.8	6	
Used a condom/had my partner use a condom	2.0	1	3.9	2	1.00
Worn covering clothes	2.0	1	5.9	3	0.500
What actions have you taken to remove mosquito breeding sites?					
Emptied/cleaned water containers	50.0	37	71.6	53	0.020
Covered water storage containers	1.4	1	17.6	13	0.002
Removed standing water	39.2	29	79.7	59	<0.001
Cleaned household environment/removed garbage	20.3	15	27.0	20	0.424
